# Fluid biomarkers for amyotrophic lateral sclerosis: a review

**DOI:** 10.1186/s13024-023-00685-6

**Published:** 2024-01-24

**Authors:** Katherine E. Irwin, Udit Sheth, Philip C. Wong, Tania F. Gendron

**Affiliations:** 1 Department of Pathology, Johns Hopkins Medicine, Baltimore, MD 21205 USA; 2 Department of Neuroscience, Johns Hopkins Medicine, Baltimore, MD 21205 USA; 3 Department of Neuroscience, Mayo Clinic, Jacksonville, FL 32224 USA; 4 Mayo Clinic Graduate School of Biomedical Sciences, Mayo Clinic, Jacksonville, FL 32224 USA

**Keywords:** Amyotrophic lateral sclerosis, Fluid biomarkers, Cerebrospinal fluid, Clinical trial, Neurofilament, Plasma, Serum, TDP-43

## Abstract

Amyotrophic lateral sclerosis (ALS) is a fatal neurodegenerative disease characterized by the loss of upper and lower motor neurons. Presently, three FDA-approved drugs are available to help slow functional decline for patients with ALS, but no cure yet exists. With an average life expectancy of only two to five years after diagnosis, there is a clear need for biomarkers to improve the care of patients with ALS and to expedite ALS treatment development. Here, we provide a review of the efforts made towards identifying diagnostic, prognostic, susceptibility/risk, and response fluid biomarkers with the intent to facilitate a more rapid and accurate ALS diagnosis, to better predict prognosis, to improve clinical trial design, and to inform interpretation of clinical trial results. Over the course of 20 + years, several promising fluid biomarker candidates for ALS have emerged. These will be discussed, as will the exciting new strategies being explored for ALS biomarker discovery and development.

## Background

Amyotrophic lateral sclerosis (ALS) is a devastating neurodegenerative disease that causes progressive loss of upper and lower motor neurons, leading to death often in just two to five years from disease onset [1]. Currently, no single test can definitively diagnose ALS. Thus, diagnosis is often delayed due to the need to rule out other neurologic diseases with similar symptoms [2, 3]. Once a diagnosis is made, three U.S. Food and Drug Administration (FDA)-approved drugs are now available to help slow functional decline or prolong life [4], but degeneration cannot be reversed, and no cure for ALS has yet been developed. Biomarkers for this disease are critically needed to improve ALS management and therapeutic development. Several categories of biomarkers exist, and many could improve ALS care and research. The FDA-National Institutes of Health (NIH) Joint Leadership Council established the BEST (Biomarkers, EndpointS, and other Tools) Resource to provide a glossary of standardized terms related to biomarkers [5], and we will use this terminology here.

One biomarker category, diagnostic biomarkers, are those that detect or confirm the presence of a disease or other condition. Effective diagnostic biomarkers for ALS would facilitate earlier identification of the disease and thus allow patients to start receiving treatment or be enrolled in clinical trials at a stage when therapeutics might be more efficacious. Diagnostic biomarkers could also potentially reveal and differentiate ALS subtypes since ALS is a clinically and pathophysiologically heterogeneous condition [6].

Instead of confirming a disease’s identity after clinical symptoms appear, susceptibility/risk biomarkers are those that indicate an individual has the potential to develop a disease when clinical manifestations of the disease are not yet present. Disease mechanisms of ALS, like many other neurodegenerative diseases, may begin months or years before symptom development [7]. Consequently, identifying biomarkers that detect these processes during the presymptomatic stage could provide a means to monitor impending disease onset in individuals with genetic mutations associated with familial ALS (fALS). However, for asymptomatic individuals without known ALS risk factors, susceptibility/risk biomarkers to predict sporadic ALS (sALS) onset would need to be measured easily and in an affordable fashion, as the relatively low incidence of sALS disfavors the use of any asymptomatic testing that carries risk or considerable expense. If susceptibility/risk biomarkers accurately identify a higher likelihood of developing ALS, preventive measures may be taken to slow or avert disease onset.

Prognostic biomarkers that predict disease severity and progression would be informative for patients with ALS. From a clinical perspective, reliable prognostic biomarkers would aid in care plan development and help direct patient expectations. For clinical trials, these biomarkers would facilitate stratification of patients based on differences in disease severity and progression so that, for example, treatment groups would comprise equal numbers of individuals who are expected to be slow or fast progressors.

Predictive biomarkers, which identify individuals who are more likely to experience a particular effect from a certain therapeutic, would also greatly benefit clinical trials. Predictive biomarkers would allow for enrichment of study populations comprising individuals for whom the treatment is more likely to be effective, thus preventing the masking of drug efficacy caused by a lack of response from individuals who are not expected to benefit from the drug. With over 50 clinical trials of disease-modifying drugs for ALS having failed [8], these biomarkers are critically needed to facilitate optimal trial design and to determine potential reasons for trial failure.

To measure the effectiveness of therapeutics in clinical trials beyond patient outcomes such as symptom relief and survival, response biomarkers can be used to show that a biological response has occurred in an individual following exposure to the therapeutic. Among response biomarkers, pharmacodynamic biomarkers indicate the molecular or physiologic activity of the therapeutic. Currently, many trials suffer from the inability to determine whether the therapeutic target was engaged as intended [8], so pharmacodynamic biomarkers related to potential treatments are essential for understanding the results of clinical trials and improving future research.

Biomarkers to serve each of these uses would significantly improve the clinical management of people with ALS and treatment development. Most likely, no single biomarker will fill each of these needs, so continued exploration of different markers is necessary. Many pathomechanisms are suggested to be involved in ALS, and these have informed current therapeutic and biomarker research. A process central to ALS that has been a focus for biomarker development is neurodegeneration, as death of both upper and lower motor neurons is characteristic of the disease [2]. Proteins that are released when neurons die, such as neurofilaments and tau, have been studied as ALS biomarkers [7, 9, 10]. Another broad process that occurs in ALS and that has been proposed as a source of biomarker candidates is inflammation, although its potential disease-driving consequences remain unclear [11, 12]. While these two processes are also involved in many other neurodegenerative diseases, TAR DNA-binding protein 43 (TDP-43) proteinopathy is a pathomechanism specific to ALS and a few other diseases [13–15]. Cytoplasmic inclusions of TDP-43 are the main pathologic hallmark of ALS [14], and much research has focused on how these aggregates may drive neuron loss [16–18]. Other research has focused on the loss of function that occurs when TDP-43 is cleared from the nucleus prior to its aggregation in the cytoplasm [19–24] or upon its formation into anisotropic intranuclear spherical shells [25]. Both these aspects of TDP-43 pathology have been leveraged for biomarker development. Other potential biomarker candidates more specific to ALS include those related to genetic mutations associated with the disease.

As biomarkers have the potential to positively impact multiple facets of ALS patient care and treatment development, substantial effort has been directed towards biomarker discovery for ALS [26]. These endeavors have benefited from the continuous growth of our knowledge of ALS pathomechanisms and advances in technologies and bioinformatics. Over the course of 20 + years, several fluid biomarker candidates for ALS have emerged, as have numerous non-fluid biomarkers ranging from clinical to radiographic markers [27–32]. Here, we provide an overview of the more promising fluid biomarkers for ALS, highlight exciting new strategies towards testing the utility of TDP-43—a protein intimately involved in ALS pathogenesis—as a reliable biomarker, and discuss present limitations and future avenues being explored for ALS biomarker discovery.

## Neurofilaments

Neurofilaments are among the most widely characterized fluid biomarkers across neurodegenerative diseases. Present only in neurons, these intermediate, filamentous proteins form part of the cytoskeletal structure and are especially abundant in myelinated axons [33]. Because they are released into the interstitial fluid during neuroaxonal injury and readily measured in cerebrospinal fluid (CSF), neurofilaments — particularly neurofilament light (NfL) and phosphorylated neurofilament heavy (pNfH) — have emerged as markers of neuronal injury and degeneration. Consequently, neurofilaments in CSF have been extensively explored as diagnostic, prognostic, susceptibility/risk, and response biomarkers for ALS. And, with the development of sensitive techniques allowing the detection of neurofilaments in blood, which is less invasively collected than CSF, similar studies examining plasma and serum neurofilaments in ALS are on the rise (Table 1).

**Table 1:** Studies evaluating neurofilaments as biomarkers for ALS/FTD

Biomarker category	Biofluid and biomarker	Author	Study cohorts
**Prognostic**	**CSF NfL**	Zetterberg et al. (2007) [34]	79 ALS patients, 40 controls
		Lu et al. (2015) [35]	38 ALS patients, 20 controls
		Steinacker et al. (2016) [36]	253 MND patients, 85 MND mimics
		Gaiani et al. (2017) [37]	94 ALS patients, 20 FTD patients, 18 ALS mimics, 44 controls
		Illan-Gala et al. (2018) [38]	86 FTD patients, 38 ALS patients, 49 controls
		Scarafino et al. (2018) [39]	85 ALS patients, 30 ALS mimics, 51 controls
		Kasai et al. (2019) [40]	Discovery cohort: 29 ALS patients, 29 controls Validation cohort: 46 ALS patients, 46 controls
		Abu-Rumeileh et al. (2020) [41]	80 ALS patients, 46 ALS mimics, 43 controls
		Thouvenot et al. (2020) [42]	207 ALS patients, 21 controls
		Brodovitch et al. (2021) [43]	20 ALS patients, 17 IPN patients
		Dreger et al. (2021) [44]	156 ALS patients, 11 ALS mimics, 56 disease controls, 15 healthy controls
		Tortelli et al. (2012) [45]	37 ALS patients, 46 neurological controls
		Vacchiano et al. (2021) [46]	171 ALS patients, 60 ALS mimics
		Gong et al. (2018) [47]	80 ALS patients, 40 controls
		Rossi et al. (2018) [48]	190 ALS patients, 130 disease controls
		Schreiber et al. (2018) [10]	89 ALS patients, 33 healthy controls
		Kojima et al. (2021) [49]	75 ALS patients
	**Blood NfL**	Lu et al. (2015) [35]	167 ALS patients, 78 controls
		Kasai et al. (2019) [40]	Discovery cohort: 29 ALS patients, 29 controls Validation cohort: 46 ALS patients, 46 controls
		Brodovitch et al. (2021) [43]	60 ALS patients, 94 controls, and 43 IPN patients
		Thompson et al. (2022) [50]	258 ALS patients, 80 neurological disease controls, 101 healthy controls
		Gille et al. (2019) [51]	149 ALS patients, 19 ALS mimics, 82 disease controls
		Verde et al. (2019) [52]	124 ALS patients, 50 healthy controls, 44 disease controls
		Vacchiano et al. (2021) [46]	171 ALS patients, 60 ALS mimics
		Gong et al. (2018) [47]	80 ALS patients, 40 controls
		De Schaepdryver et al. (2020) [53]	383 ALS patients
		Behzadi et al. (2021) [54]	234 ALS patients, 44 ALS mimics, 9 controls
	**CSF pNfH**	Gendron et al. (2017) [60]	131 ALS/FTD patients, 28 presymptomatic carries, 37 controls
		De Schaepdryver et al. (2018) [55]	85 ALS patients, 31 ALS mimics, 215 disease controls
		Simonini et al. (2021) [56]	115 ALS patients, 28 ALS mimics
		Boylan et al. (2013) [57]	20 ALS patients
		Rossi et al. (2018) [48]	190 ALS patients, 130 disease controls
		Steinacker et al. (2016) [36]	253 MND patients, 85 MND mimics
		Zecca et al. (2022) [58]	128 ALS patients, 128 controls
		Ganesalingam et al. (2011) [59]	Training cohort: 45 ALS patients, 25 disease controls, 36 healthy controls Test cohort: 26 ALS patients, 27 disease controls, 4 healthy controls
	**Blood pNfH**	De Schaepdryver et al. (2018) [55]	85 ALS patients, 31 ALS mimics, 215 disease controls
		Boylan et al. (2013) [57]	43 ALS patients
		Falzone et al. (2020) [61]	219 MND patients
		Benatar et al. (2018) [7]	17 ALS patients, 34 controls, 84 at-risk individuals, 10 phenoconverters
**Susceptibility/Risk**	**CSF NfL**	Benatar et al. (2018) [7]	17 ALS patients, 34 controls, 84 at-risk individuals, 10 phenoconverters
	**Blood NfL**	van der Ende et al. (2019) [62]	59 symptomatic mutation carriers, 149 presymptomatic mutation carriers, and 127 non-carriers
		De Schaepdryver et al. (2019) [63]	95 ALS patients, 35 MCI patients, 85 healthy controls
**Response**	**Blood NfL**	Miller et al. (2022) [64]	72 ALS patients who received tofersen, 36 ALS patients who received placebo

*ALS* amyotrophic lateral sclerosis, *bvFTD* behavioral variant frontotemporal dementia, *IPN* inflammatory peripheral neuropathies, *MCI* mild cognitive impairment, *MND* motor neuron disease

### Neurofilaments as diagnostic markers

Compared to healthy controls, NfL and pNfH concentrations in CSF, plasma or serum are elevated in ALS [9, 34–38, 40–44, 50, 52, 58–60, 65, 66] as well as other neurological diseases such as frontotemporal dementia (FTD) [37, 38, 67, 68], Alzheimer’s disease [69], stroke [70] and COVID-19 [71]. Nevertheless, neurofilament levels are often highest in patients with ALS [38, 54, 60, 65, 72, 73], likely because damage to large myelinated motor neurons, as occurs in ALS, contributes to a mass release of neurofilaments into CSF and blood.

Although increases in neurofilaments in biofluids are not disease-specific, and thus not anticipated to diagnose ALS on their own, measuring neurofilaments does provide a relatively easy means to confirm or rule out neurodegeneration in patients suspected to have ALS [73]. Neurofilament concentrations may additionally show differential diagnostic potential in clinically relevant situations such as distinguishing ALS from ALS mimic syndromes, a heterogeneous group of conditions with clinical features that initially resemble ALS (e.g., neuropathies, myelopathies, and myopathies) [74]. Studies have shown that CSF and blood neurofilaments are higher in ALS than in ALS mimics, and show utility in differentiating the two groups [36, 37, 39, 41, 43–46, 51, 52, 54–56, 66, 75, 76]. These data suggest that fluid neurofilament concentrations may facilitate a more rapid and accurate ALS diagnosis. Nevertheless, incorporating additional biomarkers involved in the pathophysiology of ALS and of ALS mimic syndromes may further improve diagnostic accuracy. For example, Brodovitch et al. showed that coupling CSF NfL with CSF intercellular adhesion molecule-1, a marker of vascular damage, and with serum interferon gamma, a marker of peripheral inflammation, better discriminated patients with ALS from those with inflammatory peripheral neuropathies than when using CSF NfL alone [43]. While these findings from relatively small cohorts remain to be validated, they do highlight that a multi-marker approach may have increased power to distinguish ALS from its mimics, and may thus help overcome present delays in accurately diagnosing ALS [77]. It must nonetheless be noted that diagnostic delays are not likely to be prevented by biomarkers alone; rather, because of the insidious nature of ALS with its slow onset and initial subtle symptoms, patients are less likely to seek timely care. Overcoming this barrier will require that clinicians and patients alike have a better understanding of early symptoms potentially representing ALS [78].

### Neurofilaments as prognostic markers

Studies have demonstrated that rises in neurofilament concentrations in patients with ALS eventually plateau within a year or so from symptom onset [35, 50, 79, 80], and that neurofilament concentrations at steady-state show promise as prognostic biomarkers. Multiple studies have reported that higher baseline neurofilament concentrations in CSF or blood associate with shorter survival [10, 34–44, 46–48, 50–54, 57, 59–61, 73, 79]. Several studies have also examined associations of neurofilament proteins with symptom severity as determined using the revised ALS Functional Rating Scale (ALSFRS-R), which has a maximum score of 48 with lower scores indicating a more severe disease stage [81]. Some studies [10, 36, 39, 45, 47, 50, 54, 73], but not all [38, 43, 52, 54, 58, 79], found that neurofilament protein concentrations inversely correlate with ALSFRS-R scores at time of diagnosis or at sample collection albeit sometimes only weakly. Additionally, several groups have shown associations of neurofilaments with actual or estimated rates of change in ALSFRS-R scores, a measure of disease progression rate [10, 35–39, 41–43, 45–54, 57, 58, 61, 79]. Using an alternate method to model disease progression, that being the D50 model, which quantifies an individual’s disease aggressiveness as the time taken in months to lose 50% function, Dreger et al. similarly found that higher CSF NfL associates with more aggressive disease [44].

As mentioned, a reliable prognosis is not only important to the patient, their family, and caregivers, it can also improve multiple aspects of clinical trial design. For instance, the heterogeneity of disease course among patients with ALS could result in different proportions of fast and slow progressors in clinical treatment arms. Using CSF or blood neurofilament concentrations as a marker of disease aggressiveness could facilitate the stratification of patients into balanced groups to reduce variability in treatment outcomes. The prognostic utility of neurofilaments, however, would be greatest when their levels are measured early in established disease; as expressed by Benatar et al., if neurofilaments are measured too late, there could be little future prognosis to forecast [78].

### Neurofilaments as susceptibility/risk biomarkers

For asymptomatic individuals with a known ALS-causing mutation, longitudinal CSF and blood neurofilament measures could inform impending symptom onset and, in so doing, improve patient care and accelerate effective therapy development. As mentioned, a major challenge in developing ALS therapies is that they are often administered months after symptom onset because of diagnostic delay. However, since pathological processes underpinning neurodegeneration occur well before first symptoms, potential therapies are expected to be more effective when initiated early in the disease course, and most effective when initiated prior to the emergence of clinical symptoms. It is thus notable that a growing body of evidence suggests that neurofilaments show promise as susceptibility/risk biomarkers given their potential to track disease progression and possibly predict the timing of clinical phenoconversion for asymptomatic individuals with a known mutation in an ALS-associated gene [e.g., superoxide dismutase 1 (*SOD1*), C9orf72-SMCR8 complex subunit (*C9orf72*), FUS RNA binding protein (*FUS)*] [7].

In a study of 84 individuals at-risk of fALS, Benatar et al. acquired longitudinal serum samples and clinical data before symptom onset, around the time symptoms developed, and in the early stages of manifest disease. In so doing, they uncovered that, in at-risk individuals who phenoconverted during the study (all but one of whom had a *SOD1* mutation), serum NfL increased in the 12 months preceding symptom onset and continued to increase for at least the six months thereafter [7]. In a subsequent study that included additional phenoconverters, Benatar et al. observed a presymptomatic increase in both serum pNfH and NfL among phenoconverters. They further found that presymptomatic increases in neurofilaments may depend on genotype given that rises in NfL were observed 6–12 months before phenoconversion in *SOD1 A4V* carriers, 3.5 years before phenoconversion in a *C9orf72* repeat expansion carrier, and 2 years before phenoconversion in a *FUS c.521del6* carrier [80].

Findings from the prospective longitudinal studies above provide insight as to when, in the natural history of fALS, neurofilament concentrations rise. Further corroborating that blood neurofilaments increase in at-risk individuals nearing phenoconversion are studies of presymptomatic individuals with mutations in *C9orf72* (associated with ALS and FTD), or in *GRN* or *MAPT* (associated with FTD). For example, Gendron et al. found that, compared to plasma NfL concentrations in clinically normal controls or presymptomatic mutation carriers who did not phenoconvert within one year of baseline, baseline plasma NfL and rates of NfL change were higher in presymptomatic carriers before phenoconversion [68]. Similarly, others have reported higher baseline serum NfL or higher rates of change in plasma NfL in presymptomatic phenoconverters than in non-converters [62, 82]. The rise in neurofilaments prior to ALS symptom onset is not unique to individuals with fALS (though it is certainly easier to monitor in this at-risk population). For example, a matched case–control study nested in three large prospective US cohorts (the Nurses’ Health Study, the Health Professionals Follow-up Study, and the Multiethnic Cohort Study) revealed that plasma NfL is elevated in individuals who were later diagnosed with ALS, with increases generally being observed 12–24 months before an ALS diagnosis [83]. It was also reported that serum pNfH is elevated well before the time individuals were diagnosed with sALS [63]. In this same study, five of the ten patients with ALS from whom blood was collected presymptomatically had elevated serum pNfH based on a pre-defined cutoff, and three of these individuals had elevated pNfH as early as 26 months before symptom onset.

In aggregate, these findings indicate that blood NfL could facilitate the identification of presymptomatic mutation carriers approaching phenoconversion; nevertheless, it is likely that the addition of traits, such as age and mutation status, would better approximate when clinically manifest ALS will occur. This notion is supported by disease progression models for familial FTD [84], and the same is expected for ALS. Biomarkers of phenoconversion would facilitate the identification of suitable presymptomatic individuals to participate in clinical trials designed to prevent or delay symptom onset and progression. Indeed, if there is no susceptibility/risk biomarker to forecast impending phenoconversion, recruiting presymptomatic mutation carriers to prevention treatment trials is challenging. Notably, the use of plasma NfL in predicting phenoconversion in presymptomatic carriers of *SOD1* variants associated with high or complete penetrance and rapid disease progression has enabled the first interventional trial in presymptomatic ALS [85]. The study (NCT04856982), called ATLAS, is a randomized, placebo-controlled phase 3 trial with a longitudinal natural history run-in designed to evaluate whether intrathecal injections of BIIB067 (tofersen), an antisense oligonucleotide (ASO) designed to reduce SOD1 synthesis [86, 87], can delay the emergence of clinically manifest ALS. During the no intervention natural history run-in phase (Part A) of this study, presymptomatic carriers of *SOD1* variants with plasma NfL concentrations below the pre-defined threshold are enrolled, and plasma NfL levels are then measured once every 28 days. Presymptomatic participants from Part A whose plasma NfL concentrations rise above the threshold may be enrolled in Part B, and be administered tofersen or placebo on days 1, 15, 29 and every 28 days thereafter for up to two years. The primary endpoint of Part B is the percentage of participants in whom clinically manifest ALS emerges within one year from baseline. Secondary endpoints include changes from baseline to end of study in ALSFRS-R total score, percent predicted slow vital capacity (SVC; a spirometry measurement capturing the volume of air produced from maximal inspiration to maximal expiration without forced or rapid effort), need for ventilation, death, and total CSF SOD1 and plasma NfL concentrations [85]. Of note, whereas plasma NfL is initially utilized to select appropriate study participants for this clinical trial, as discussed below, its second use is to monitor treatment effect.

### Neurofilaments as response markers

In addition to serving as susceptibility/risk and prognostic biomarkers, CSF and blood neurofilaments may serve as response biomarkers. Although there is presently insufficient data supporting that neurofilament levels are a substitute for how well a patient feels or functions, now that clinical trials for potential ALS therapies often include neurofilament measures as secondary (and even primary) endpoints, data from these trials are likely to inform whether neurofilaments can be considered as a reasonably likely surrogate endpoint for clinical benefit.

Neurofilaments may also serve as pharmacodynamic biomarkers in that decreases in CSF or blood neurofilaments in response to potential therapeutics could indicate biological activity of the drug being tested. For example, in a double-blind, randomized, placebo-controlled phase 3 clinical trial named VALOR (NCT02623699), the efficacy and safety of the *SOD1*-targeting ASO, tofersen, was tested in symptomatic patients with *SOD1* ALS [64]. In this study, 72 participants received tofersen, and 36 received placebo. Although statistical significance in participants receiving tofersen was not met for the primary endpoint, that being a change in ALSFRS-R score from baseline to week 28, trends of reduced disease progression across multiple secondary and exploratory endpoints were observed as were robust decreases in CSF SOD1 and plasma NfL. Once the VALOR study was completed, 63 participants who originally received tofersen and 32 participants who originally received placebo were enrolled in an open-label extension and received tofersen. Participants who received tofersen for 52 weeks (28 weeks during the VALOR study and 24 weeks during the open-label extension) displayed consistent slowing of disease progression across endpoints and sustained decreases in CSF SOD1 and plasma NfL [64]. While the extension phase is not yet complete, these early findings suggest that NfL, and SOD1, show promise as a pharmacodynamic biomarkers.

### Limitations and future perspectives of neurofilament protein biomarkers

In aggregate, ample data suggest that CSF and blood neurofilament proteins show utility across multiple biomarker categories. Nevertheless, one must be cognizant of their limitations. As noted, measures of neurofilaments can inform the presence of neuronal injury and degeneration in ALS and other neurological diseases. But, on their own, neurofilaments cannot be used to definitively diagnose ALS. Neurofilaments do show promise in predicting impending disease onset in individuals at known risk of ALS, with longitudinal increases in neurofilaments occurring prior to clinically manifest ALS. Of note, the timing of such increases varies by genotype, and it has been posited that the duration of the presymptomatic phase is proportional to the symptomatic phase—meaning that the presymptomatic phase is longer in patients with slowly progressing disease, and shorter in patients with rapidly progressing disease [80]. While additional investigations are needed to test the latter, the study of phenoconverters is hampered by small cohort sizes, and necessarily long observation periods. Nevertheless, the ability to measure biofluid neurofilaments in presymptomatic mutation carriers provides a means to gauge impending phenoconversion, thus enabling the recruitment of presymptomatic mutation carriers to prevention treatment trials. Perhaps the greatest potential of neurofilaments is their prognostic utility. Despite discrepancies among some findings as noted above, which may be caused by differences in cohort sizes, matrices (i.e., CSF, plasma, serum), neurofilament types (i.e., NfL or pNfH), assay sensitivity, and analytical rigor, neurofilaments are leading prognostic biomarkers for ALS. Determining both the ideal assay with which to measure neurofilaments and the ideal neurofilament type, as well as establishing normative values and identifying potential confounders (e.g., chronic kidney disease and acute neuroaxonal injury) will improve reproducibility. Moreover, the advent of machine learning and amalgamating neurofilament data with genetic, clinical, digital, imaging, and other molecular marker data are anticipated to provide more efficient means to diagnose, prognose and treat ALS.

## SOD1 as a pharmacodynamic marker

As mentioned above, lowering SOD1 is being investigated as a therapeutic for *SOD1* ALS given that the pathological misfolding and aggregation of mutant SOD1 are believed to underlie its toxicity [88]. In preclinical studies using rodents expressing human *SOD1 G93A* or using non-human primates, *SOD1*-targeting ASOs decreased *SOD1* mRNA and protein in the central nervous system (CNS), delayed symptom onset, and/or slowed disease progression [86, 89, 90]. The ASO additionally decreased CSF SOD1 in rats and non-human primates suggesting that CSF SOD1 has utility as a pharmacodynamic marker [86, 90]. The use of CSF SOD1 as a marker of target engagement is supported by data from the aforementioned VALOR study showing that tofersen administration decreases CSF SOD1.

Though a former study of patients with sALS found no association between CSF SOD1 and disease characteristics [90], it will be of great interest to examine, once the tofersen extension phase is complete, whether larger decreases in CSF SOD1 associate with greater improvements in clinical end-points in patients with *SOD1* ALS. In this manner, information may be gleaned as to whether CSF SOD1 shows potential as a surrogate endpoint biomarker to predict clinical benefit*.*

## Dipeptide repeat proteins as pharmacodynamic markers

G_4_C_2_ hexanucleotide repeat expansions in the *C9orf72* gene are the most common known genetic cause of ALS and FTD [91, 92]. These bidirectionally transcribed expansions cause the accumulation of G_4_C_2_ or G_2_C_4_ repeat RNA, and the production of five aggregation-prone dipeptide repeat (DPR) proteins. Nuclear foci formed of the repeat RNA and DPR proteins are thought to contribute to disease pathogenesis, as is loss of C9orf72 function. Discovery of these traits specific to *C9orf72*-associated ALS and FTD (c9ALS/FTD) brought to light new therapeutic targets, chief among them, repeat RNA and DPR proteins formed thereof. This quickly prompted early investigations to identify approaches to neutralize or degrade the repeat RNA [93–99], and to establish means to monitor whether the therapeutic strategies being tested have the intended outcomes [97]. With the latter in mind, and with the expectation that lowering repeat RNA would attenuate DPR protein production, an immunoassay was developed to measure poly(GP) DPR proteins in preclinical models and in CSF from *C9orf72* repeat expansion carriers [97]. This study engendered a number of subsequent investigations on the prognostic and pharmacodynamic potential of poly(GP) and other DPR proteins. Poly(GP), poly(GA) and poly(GR) were detected in CSF from asymptomatic and symptomatic *C9orf72* repeat expansion carriers, with levels being generally similar between these two groups, but were not detected in CSF from individuals with no *C9orf72* repeat expansion [93, 100–103]. Levels of CSF DPR proteins in symptomatic *C9orf72* expansion carriers did not associate with clinical traits such as age at disease onset, ALSFRS-R score, rate of decline of ALSFRS-R score or survival, nor with NfL concentrations [93, 100–103]. Despite their lack of prognostic utility, ample evidence from preclinical models supports the use of CSF DPR proteins as pharmacodynamic biomarkers. For example, treating c9ALS/FTD mouse models with an ASO targeting G_4_C_2_ repeat-containing transcripts resulted in sustained decreases in brain, spinal cord and CSF DPR proteins [93, 98, 99, 104, 105]. What is more, repeated intrathecal delivery of a G_4_C_2_ repeat-targeting ASO to a *C9orf72* repeat expansion carrier significantly decreased CSF poly(GP), poly(GA) and poly(GR) [103, 105].

The data above support the use of DPR proteins as preclinical and clinical pharmacodynamic biomarkers for therapeutic approaches targeting G_4_C_2_ repeat RNA. In March 2022, Biogen and Ionis Pharmaceuticals announced that BIIB078, an investigational ASO for *C9orf72*-associated ALS that degrades G_4_C_2_ expansion-containing mRNA, did not show clinical benefit in their phase 1 clinical trial (NCT03626012), which has since been discontinued. Whether BIIB078 decreased CSF DPR proteins in trial participants has not been publicly reported. This information, along with data on DPR protein levels from similar clinical trials (e.g., the Wave Life Sciences NCT04931862 trial evaluating intrathecal WVE-004, an ASO targeting repeat-containing pre-mRNA variants in patients with c9ALS/FTD), would facilitate the interpretation of trial results and inform whether alternative therapeutic approaches for c9ALS/FTD should be considered.

## TDP-43-related fluid biomarkers

While neurofilaments are elevated in numerous neurological diseases, TDP-43 proteinopathy is the main pathological hallmark of ALS-FTD [14] and occurs in a few other neurodegenerative diseases, such as limbic-predominant age-related TDP-43 encephalopathy (LATE) [15, 106] and Alzheimer’s disease [106–110]. TDP-43 is a ubiquitously expressed DNA/RNA-binding protein that is mainly localized to the nucleus, but, in up to 97% of cases of ALS, it is cleared from the nuclei of neurons and glia and forms pathological inclusions in the cytoplasm of some of these cells [14, 111, 112]. These aggregates, composed in part of ubiquitinated and hyperphosphorylated TDP-43 as well as truncated C-terminal TDP-43 fragments [14, 112], are believed to cause toxic gains-of-function [16–18]. Other studies have highlighted detrimental loss-of-function mechanisms that occur upon nuclear TDP-43 depletion [23, 24]. Given the centrality of TDP-43 pathology in ALS, this protein has been targeted for biomarker development (Table 2). While the affected neurons and glia of the brain and spinal cord cannot be accessed directly in living patients, the functional and structural abnormalities associated with TDP-43 nuclear clearance and cytoplasmic aggregation suggest that these processes may lead to molecular changes that are reflected in accessible biofluids. Indeed, hypothesizing that TDP-43 produced in the CNS may be released into CSF or blood, and differ between individuals with or without ALS, several groups measured TDP-43 in these fluids. Some studies examined full-length TDP-43 in CSF or blood as an ALS biomarker [40, 49, 113–117], while others focused on disease-associated TDP-43 modifications [118, 119] or pathological TDP-43 fragments [40, 49].

**Table 2:** Studies evaluating TDP-43 as a biomarker for ALS/FTD

Biospecimen/biomarker	Author	Population
**CSF TDP-43**	Steinacker et al. (2008) [113]	12 FTD patients, 15 ALS patients, 9 ALS-FTD patients, 3 ALS patients with additional signs of frontal disinhibition, 13 controls
	Kasai et al. (2009) [114]	30 ALS patients, 29 controls
	Hosokawa et al. (2014) [115]	13 ALS patients, 7 GBS patients
	Noto et al. (2011) [116]	27 ALS patients, 50 neurodegenerative or inflammatory controls
	Kasai et al. (2019) [40]	Discovery cohort: 29 ALS patients; 29 controls Validation cohort: 46 ALS patients; 46 neuromuscular disease controls
	Ren et al. (2021) [119]	69 ALS patients, 59 healthy controls
	Majumder et al. (2018) [120]	Meta-analysis of 7 studies
	Kojima et al. (2021) [49]	75 ALS patients
**Plasma TDP-43**	Kasai et al. (2019) [40]	Discovery cohort: 29 ALS patients; 29 controls Validation cohort: 46 ALS patients; 46 neuromuscular disease controls
	Verstraete et al. (2012) [117]	219 ALS patients, 100 controls
	Ren et al. (2021) [119]	69 ALS patients, 59 healthy controls
	Kojima et al. (2021) [49]	75 ALS patients
**TDP-43 scFv reactivity patterns in plasma**	Williams et al. (2017) [121]	4 sporadic ALS patients, 4 *C9orf72* ALS patients, 3 controls
**TDP-43 secondary structure distribution in CSF**	Beyer et al. (2021) [118]	36 ALS patients, 30 Parkinson’s disease patients, 24 further controls
**Naturally occurring TDP-43 autoantibodies in plasma**	Nielsen et al. (2021) [122]	30 ALS patients, 51 healthy controls
**CSF pTDP-43**	Ren et al. (2021) [119]	69 ALS patients, 59 healthy controls
**Plasma pTDP-43**	Ren et al. (2021) [119]	69 ALS patients, 59 healthy controls
**CSF TDP-43 and NfL or tau**	Kasai et al. (2019) [40]	Discovery cohort: 29 ALS patients; 29 controls Validation cohort: 46 ALS patients; 46 neuromuscular disease controls
	Bourbouli et al. (2017) [123]	32 ALS patients, 51 FTD patients, 17 healthy controls

*ALS* amyotrophic lateral sclerosis, *FTD* frontotemporal dementia, *GBS* Guillain-Barré syndrome, *NfL* neurofilament light, *sALS* sporadic amyotrophic lateral sclerosis

### Quantification of TDP-43 in CSF and plasma

TDP-43, a 414-residue protein [124], was first analyzed as a potential CSF biomarker for ALS in 2008 [113]. Immunoblot analysis using a polyclonal antibody against TDP-43 residues 1–260 revealed higher levels of a 45-kilodalton (kDa) band in CSF of patients with ALS as compared to controls. Soon after, Kasai et al. quantified TDP-43 in CSF from individuals with ALS by enzyme-linked immunosorbent assay (ELISA) [114]. When using the same capture and detection antibodies employed in their ELISA to instead immunocapture TDP-43 from CSF and then probe for TDP-43 by immunoblot analysis, a 43-kDa band was detected, suggesting that the ELISA predominately detects full-length TDP-43. The group observed higher CSF TDP-43 measured by ELISA in individuals with sALS compared to age-matched healthy or neurological disease controls. Additionally, they found significantly higher TDP-43 in CSF when it was collected from patients with ALS within 10 months of disease onset compared to later time-points, suggesting that elevated CSF TDP-43 may show utility as an early marker of disease.

Hosokawa et al. also developed a TDP-43 ELISA with the same coating and detection antibodies as above [115]. It was used to determine whether CSF TDP-43 could distinguish patients with ALS from those with Guillain-Barré syndrome (GBS), an inflammatory peripheral neuropathy that can also affect motor neurons [125]. CSF TDP-43 was significantly higher in ALS compared to GBS, and a cutoff value of 1.16 ng/mL produced a sensitivity of 84.6% and a specificity of 71.4%. The negative predictive value was 62.5%, while the positive predictive value was 83.3%, suggesting that these CSF TDP-43 measurements alone may not meet clinical application standards. Contrary to the study by Kasai et al. [114], CSF TDP-43 did not associate with disease duration in patients with ALS, nor did it associate with age [115].

Also using a TDP-43 ELISA, CSF TDP-43 was found to be increased in ALS compared to neurodegenerative disease and inflammatory neurological disease controls [116], consistent with prior data [114, 115]. A cutoff value of 27.9 ng/mL showed a sensitivity of 59.3% and a specificity of 96%, suggesting that measures below the cutoff may not rule out an ALS diagnosis, but a positive test could help distinguish ALS from other neurological diseases. This study, however, did not include ALS mimics, which are more difficult to clinically distinguish from patients with ALS. TDP-43 in CSF was higher in the ALS group than the control group, and patients with ALS with higher CSF TDP-43 survived significantly longer than those with lower levels. While this suggests the utility of CSF TDP-43 as a prognostic biomarker, these findings were generated from a small cohort of 27 patients with ALS and have not since been replicated. In a later study, a commercial assay produced by Quanterix that is expected to detect full-length and pathologically truncated TDP-43 was employed [40]. Both CSF and plasma TDP-43 were higher in patients with ALS compared to controls in the discovery cohort, but, in the validation cohort, only CSF TDP-43 was elevated in ALS compared to controls. Moreover, survival did not associate with CSF TDP-43 levels in either cohort nor in both cohorts combined [40]. In a study comprising 219 patients with ALS and 100 age- and sex-matched healthy controls, plasma TDP-43 measured by ELISA was higher in the ALS cohort [117]. However, TDP-43 concentrations were above the assay detection limit in only 28% of these patients vs. 21% of controls, suggesting the need for a more sensitive assay. Consistent with findings by Kasai et al., who measured CSF TDP-43 using a Quanterix assay [40], this study found no association between plasma TDP-43 and survival. Additionally, although Kasai et al. found higher CSF TDP-43 in individuals within 10 months of ALS onset [114], plasma TDP-43 did not associate with disease duration in this large study, but it did associate with age in patients with ALS and in controls [117].

Using two commercial ELISAs, Ren and colleagues analyzed total TDP-43 and phosphorylated TDP-43 (pTDP-43) in CSF and plasma [119]. Both plasma TDP-43 and pTDP-43, but not CSF TDP-43 or pTDP-43, were significantly elevated in patients with ALS compared to healthy controls. Likewise, plasma pTDP-43/TDP-43 ratios, but not CSF pTDP-43/TDP-43 ratios, were significantly lower in patients with ALS compared to controls. Plasma TDP-43 provided an area under the receiver operating characteristic curve (AUC) of 0.924 with a sensitivity of 91.3% and a specificity of 91.5%, while plasma pTDP-43 provided an AUC of 0.765, a sensitivity of 82.6%, and a specificity of 67.8%.

### Combination of TDP-43 with other fluid biomarkers

As indicated above, several studies observed higher CSF or plasma TDP-43 in individuals with ALS. A meta-analysis of seven such studies demonstrated significantly higher CSF TDP-43 in patients with ALS with an effect size of 0.64 (95% CI [0.1–1.19], *p* = 0.02) [120]. However, the sensitivity and specificity of these findings varied widely prompting some groups to evaluate whether combining TDP-43 with other fluid biomarkers would improve diagnostic performance. Indeed, combining CSF TDP-43 and NfL to distinguish patients with ALS from controls yielded a higher AUC than either biomarker alone [40]. Furthermore, a study aiming to distinguish individuals with ALS from controls found that CSF TDP-43, total tau (T_T_), and tau phosphorylated at threonine 181 (T_P-181_) in a ratio of (TDP-43 × T_T_)/T_P-181_ increased the sensitivity from 0.72 to 1.00, the specificity from 0.77 to 0.92, and the AUC from 0.75 to 0.97 compared to measuring TDP-43 alone [123]. No specific combination of biomarkers was identified as being superior.

### TDP-43 quantification and functional measures

Some studies examined associations of CSF or blood TDP-43 with functional measures in patients with ALS. Whereas CSF TDP-43 did not associate with the functional measures tested, Kojima et al. observed that higher plasma TDP-43 associated with lower split hand index (SI) [49], a measure of hand muscle atrophy that is significantly decreased in patients with ALS compared to individuals with mimic disorders [126]. Conversely and counterintuitively, higher plasma TDP-43 associated with higher ALSFRS-R scores reflecting better global function, and also with higher SVC, which associates with better respiratory function [49, 127] and with longer survival [128]. In contrast to these results, Ren and colleagues observed that higher CSF pTDP-43 correlated with worse ALSFRS-R scores. However, they also found that higher plasma TDP-43 correlated with a more prolonged time to generalization (i.e., the time of symptoms spreading from bulbar or spinal localization to both), reflecting slower disease progression [119]. The underlying reasons for these divergent findings require further investigation but may result from clinical variations based on disease stage.

### Other approaches to TDP-43 biomarker development

Most studies have employed standard ELISA protocols using CSF or plasma to assess the utility of TDP-43 as a fluid biomarker; however, several groups adopted different approaches. For example, one group developed an atomic force microscopy-based biopanning protocol and isolated single chain antibody fragments (scFvs) that preferentially bound TDP-43 variants in ALS brain tissue compared to FTD and healthy brain tissues [129]. They established an ELISA with these scFvs and found that nine scFvs reacted with all sALS plasma samples while none reacted with plasma from controls [121]. Plasma from different patients with ALS displayed different reactivity patterns, suggesting that various TDP-43 pathology profiles exist. Further study is needed to reveal whether personalized biomarker profiles of TDP-43 variants could benefit patients and provide clinical utility.

Rather than examining TDP-43 levels, a study was undertaken to evaluate the secondary structure distribution of TDP-43 in CSF [118]. Immuno-infrared sensor technology was employed to measure TDP-43 misfolding, and these measurements discriminated individuals with ALS from individuals with Parkinson’s disease with a sensitivity of 89% and specificity of 77%, and from patients with other neurological diseases with a sensitivity of 89% and specificity of 83%. Additionally, measurements indicative of greater misfolding were seen in patients with fast progressing ALS (those having ALSFRS-R scores that decreased ≥ 0.5 points per month), indicating potential prognostic ability of TDP-43 secondary structure analysis.

Yet another study investigated naturally occurring autoantibodies (NAbs) against TDP-43 in plasma of 30 patients with ALS and 51 healthy controls [122]. ELISA competition assays were used to assess the avidity/affinity of NAbs, and this revealed that the proportion of high-avidity/affinity anti-TDP-43 NAbs in plasma was significantly decreased in ALS. Avidity/affinity of NAbs from plasma sampled shortly after disease onset correlated with disease duration, with individuals having reduced fractions of high avidity/affinity NAbs experiencing shorter survival times. In addition to the avidity/affinity of NAbs, the immunoglobulin (Ig) types of anti-TDP-43 NAbs in plasma were analyzed. Significantly lower relative levels of IgG3 and IgM, and higher levels of IgG4 NAbs, were found in plasma from patients with ALS compared to controls. Higher relative levels of anti-TDP-43 IgM and lower relative levels of anti-TDP-43 IgG4 correlated with a longer survival in patients with ALS. Thus, both avidity/affinity and Ig class of plasma anti-TDP-43 NAbs may provide diagnostic and prognostic information in ALS, but further study is needed.

### Limitations of current TDP-43-related biomarkers

TDP-43 levels, structure, and autoantibodies in CSF or blood have been assessed as potential biomarkers for ALS. While TDP-43 seems a likely biomarker candidate due to the centrality of TDP-43 pathology in ALS, these efforts have faced several limitations. Multiple antibodies with different sensitivities and specificities have been employed to detect various TDP-43 species, making comparisons among studies difficult. In addition, given that TDP-43 is ubiquitously expressed, whether the presumably soluble TDP-43 detected in biofluids represents TDP-43 originating from the CNS is not known. Furthermore, if the TDP-43 detected in CSF or blood does originate from affected cells in the CNS, the interpretation of TDP-43 changes remains uncertain; for instance, increases in TDP-43 in these biofluids could result from increased neurodegeneration and the release of proteins, including TDP-43, from damaged neurons, while decreases in TDP-43 may be caused by its sequestration in cytoplasmic aggregates. These factors, coupled with the ambiguous nature and extent of the exchange of TDP-43 between CSF and plasma hamper our understanding of TDP-43 measurements and their application in the clinical setting. Indeed, since higher plasma TDP-43 associated with better ALSFRS-R and SVC measures but with worse SI index values [49], the relationship of plasma TDP-43 to functional outcomes seems complex. While such findings could indicate that plasma TDP-43 levels change throughout disease progression, a study on a small cohort of patients with ALS with longitudinally collected plasma found plasma TDP-43 levels to be stable in five of the six patients [117]. More comprehensive cross-sectional and longitudinal studies are required to better decipher the relationship of CSF and plasma TDP-43 with clinical and functional traits in patients with ALS.

Overall, while some interesting relationships of CSF or plasma TDP-43 and functional measures have emerged, and many studies have shown elevated TDP-43 levels in these fluids in patients with ALS compared to controls, findings from these studies may not translate to clinical utility at the level of individual patients. Additionally, as the pathological underpinnings of the TDP-43 measured in biofluids are unclear, the utility of TDP-43 as a response biomarker for clinical trials is currently limited.

### Cryptic exon-encoded neoepitopes as TDP-43-related biomarkers

Because the disease relevance of CSF and plasma TDP-43 remains puzzling, RNA targets related to TDP-43 function are being investigated as an exciting alternative approach to track TDP-43 anomalies. As mentioned previously, loss of TDP-43 function is believed to play a key role in ALS pathogenesis [19–23, 130]. When TDP-43 is cleared from the nucleus in ALS, it fails to serve its role as a splicing repressor, leading to the incorporation of nonconserved cryptic exons during RNA splicing [23].

Evidence suggests that cryptic exons resulting from TDP-43 loss of function play important roles in ALS pathogenesis. Loss of TDP-43 triggers inclusion of a cryptic exon in stathmin 2 (*STMN2*), which leads to premature polyadenylation of the transcript and reduced *STMN2* expression [20]. Rescue of STMN2 expression in TDP-43-depleted motor neurons is sufficient to restore neurite outgrowth and axon regeneration, suggesting the significance of cryptic exon-mediated loss of STMN2 for motor neuron vulnerability [19, 20]. Additionally, unc-13 homolog A (*UNC13A*) variants, which are among the strongest genetic risk factors for sporadic ALS and FTD, were shown to harbor a cryptic exon [21, 22]. Single nucleotide polymorphisms in *UNC13A* exacerbate its cryptic splicing in the context of TDP-43 dysfunction, linking genetic risk for ALS with TDP-43 dysfunction [21, 22]. Deficiencies in the function of TDP-43 as a splicing repressor thus appear to be mechanistically important in ALS.

Whether a small number of TDP-43-related cryptic exons accounts for the majority of motor neuron death in ALS, or whether many cryptic exons play roles in ALS pathogenesis, is not presently known. Nevertheless, monitoring cryptic exon products could reveal the state of TDP-43 dysfunction and thus represent promising biomarker candidates. Indeed, one group observed that some TDP-43-dependent cryptic exons are incorporated in-frame; hypothesizing that these neoantigens could be detected in patient biofluids and used as biomarkers, the group sought to develop antibodies against cryptic exon-encoded peptides [131]. To do so, they availed a published and robust transcriptomic dataset generated from motor neurons depleted of TDP-43 [19]. In this manner, candidate in-frame cryptic exons were identified based on their expression in ALS-affected cell types. Cryptic targets in ubiquitously expressed genes with relevance to other TDP-43-related diseases, such as inclusion body myositis, were also selected [132, 133]. AlphaFold protein structure prediction software was used to identify in-frame cryptic exons, expressed either in the CNS or ubiquitously, that produced immunogenic epitopes without significantly disrupting native protein conformation [134]. Several cryptic exon-encoded peptides were then used to generate novel monoclonal antibodies, allowing a sensitive sandwich immunoassay to be developed that specifically detects the cryptic exon-encoded peptide within Hepatoma-Derived Growth Factor-Like Protein 2 (HDGFL2) in CSF from individuals with c9ALS/FTD [131]. Importantly, cryptic HDGFL2 was elevated in CSF from both symptomatic and presymptomatic *C9orf72* mutation carriers compared to controls. Additionally, cryptic HDGFL2 levels were higher earlier in disease. This study showed that CSF cryptic HDGFL2 levels may rise presymptomatically, earlier than neurofilament levels, and subsequently decrease during symptomatic disease progression. Meanwhile, CSF NfL and pNfH levels tend to increase closer to symptom onset and continue to increase during the early years of symptomatic disease [7, 80]. These cryptic HDGFL2 data provide evidence that TDP-43 dysfunction occurs presymptomatically and suggest that cryptic peptides could have utility as early diagnostic biomarkers, which would facilitate earlier therapeutic intervention for patients. Additionally, these observations suggest that analyzing CSF cryptic peptide and neurofilament biomarkers together could afford prognostic utility due to their differing temporal profiles.

Another group provided a valuable contribution to the study of cryptic peptides as biomarkers by employing a targeted mass spectrometry approach to detect cryptic peptides in CSF from 15 patients with ALS/FTD [135]. Notably, this led to the detection of peptides mapping to 13 cryptic exons, including that of HDGFL2. Ten of these peptides were identified in > 80% of the patients [135]. This targeted mass spectrometry approach holds promise for identifying a comprehensive set of TDP-43 cryptic targets and other proteomic changes occurring during early stages of ALS, and for defining the natural history of familial ALS [136].

Studies assessing larger cohorts of familial and sporadic ALS and disease controls will be important for determining the diagnostic utility of cryptic peptide biomarkers. These biomarkers should be analyzed in conjunction with clinical signs and other biomarkers such as neurofilaments to establish the most robust criteria for early ALS diagnosis. If immunoassays against other cryptic peptides are developed, the dynamics of various cryptic peptides could be measured throughout the disease course, possibly providing prognostic information. Extensive longitudinal studies in familial ALS, including in the presymptomatic stage, and in sporadic ALS are warranted to elucidate relationships between changes in levels of different cryptic peptides over time and disease progression, such as changes in ALSFRS-R score over time. As cryptic peptides denote TDP-43 dysfunction, these biomarkers could additionally serve as important response biomarkers for therapeutic agents that are developed to restore TDP-43 function. Measuring cryptic peptide abundance thus offers a promising new avenue for biomarker research. Improving upon the sensitivity of these newly developed cryptic peptide assays, characterizing the temporal profile of additional cryptic peptide candidates, and developing blood-based cryptic peptide assays will be important future steps. Due to the involvement of TDP-43 dysfunction in other diseases such as Alzheimer’s disease [106, 108–110, 137], LATE [15, 106], and inclusion body myositis [132, 133], the utility of these cryptic peptide biomarkers could extend beyond ALS and FTD.

## Future of biomarkers

Neurodegenerative diseases can rarely be reduced to a single pathogenic process, and ALS is no exception. Beyond neuronal dysfunction and death, ALS involves non-neuronal cells and processes such as neuroinflammation [12, 138] and dysregulated metabolism [139–142], among others, that may too yield putative biomarkers. For example, CSF chitinases have emerged as markers of neuroinflammation in patients with ALS with higher CSF CHIT1, CHI3L1 and CHI3L2 associating with faster disease progression, greater cognitive dysfunction and/or shorter survival [143–147]. Similarly, S100 Calcium Binding Protein B (S100B), a glial protein and marker of astrogliosis, also shows prognostic potential for patients with ALS. Lower CSF S100B concentrations were found to associate with a better survival in patients with ALS [148]; however, serum S100B demonstrates only minor prognostic value in comparison to serum NfL [149]. Thus, as pathomechanisms evolve throughout the course of disease, a panel of different types of biomarkers are expected to provide more dynamic and deep insight into ALS than a single measure, especially when seeking understanding of clinical progression or therapeutic response at the level of individual patients. Although the present review focused on fluid biomarkers, this panel would ideally include fluid and non-fluid biomarkers that are already under examination and nearing clinical translation and would benefit from novel putative biomarkers discovered through the ALS field’s relentless quest to further our understanding of this devastating disease. Below, we provide an array of examples of potential fluid and non-fluid biomarkers for ALS.

Exosomes, a type of extracellular vesicle, have attracted interest as new targets for biomarker development. Exosomes can be extracted from CSF or blood, and their contents examined for proteins, nucleic acids, lipids, or other molecules that can be measured as biomarkers [150]. Several reports have already proposed new biomarkers for ALS from such studies [151–155]. One study isolated neuron-derived extracellular vesicles from plasma and found 30 differentially expressed microRNAs (miRNAs) in patients with ALS compared to controls [156], while another study observed that the lipid composition of large EVs in plasma differed between patients with ALS and healthy controls [157], suggesting that extravesical vesicle content could inform the diagnosis, and potentially the prognosis, of ALS. The ability to select exosomes derived from specific cell types improves upon the inability of many other CSF or blood-based assays to determine the origin of the analyte of interest.

Noncoding RNAs (ncRNAs) such as miRNA may serve as a future focus of biomarker development. High-quality libraries can now be generated from small amounts of RNA, and RNA-sequencing may allow for screening of ncRNA biomarkers [158]. A few studies have already employed RNA-sequencing to detect differentially expressed miRNAs in CSF [159] and blood [160] of individuals with ALS, but further study is needed to identify reliable patterns. One clinical trial (NCT03088839) investigating circulating miRNA-218 as a potential biomarker for ALS is underway [161].

In addition to fluid biomarkers, non-fluid biomarkers of ALS are being investigated [162]. For instance, electrophysiological techniques that estimate the number of motor units in a muscle were found to track with disease progression in ALS [163–165]. Moreover, structural and functional neuroimaging biomarkers may be useful for ALS assessment. Several studies employing different imaging techniques have faced limitations of inconsistent findings or variable accuracy [166, 167]. Some studies, though, have suggested potential utility of neuroimaging biomarkers. For example, one study showed associations between the spatial extent of brain atrophy measured by magnetic resonance imaging and disease progression in ALS, suggesting potential prognostic utility of neuroimaging [168]. Larger-scale studies are needed to substantiate findings generated from prior small cohorts. Technological advancements that further improve the sensitivity of different imaging modalities will increase the ability of these techniques to serve as biomarkers in the clinic or for research.

Technological advancement has also facilitated the development of digital biomarkers. The FDA defines a digital biomarker as “a characteristic or set of characteristics, collected from digital health technologies, that is measured as an indicator of normal biological processes, pathogenic processes, or responses to an exposure or intervention, including therapeutic interventions” [169]. Various devices can collect digital biomarker data measuring gait speed/symmetry, stride length, finger tapping speed, eye movements, facial expressions, reaction time, spatial memory, and many other variables. These measurements can be collected from patients longitudinally at home, generating datasets richer than are possible from studies requiring in-person visits. Such datasets, especially those combining many different biomarkers, are likely to reveal clinically subtle disease signatures that can be detected across ALS progression. Research on digital biomarkers for ALS is nascent, so as the digital health technologies enabling these biomarkers continue to advance, large-scale studies evaluating the effectiveness and disease relevance of these tools are needed [170].

Both phenotypic and genotypic data will be useful for guiding ALS treatment. Genome-wide genetic data can be combined computationally into a polygenic risk score to predict an individual’s inherited susceptibility to ALS [171]. Polygenic risk scores for ALS have shown significant associations with verbal-numerical reasoning in one study [172], but further research is needed to determine whether other associations between genetics and clinical features of ALS exist beyond the handful of mutations already known to cause ALS. Identification of genetic profiles that predict response to different therapeutics would be beneficial for stratifying patients in clinical trials.

While many biomarkers for ALS are under investigation, few have been extensively studied. As such, identifying the best biomarkers will be an ongoing endeavor as new biomarker candidates are continuously being investigated and in various stages of validation. Fluid biomarker development will benefit from the increased use of blood-based vs. CSF-based assays. With the greater accessibility of blood samples, more extensive longitudinal studies in larger and more diverse cohorts will be feasible, offering richer datasets for analysis. Technological advancements are additionally expected to improve fluid biomarker assay sensitivity as well as expand newer biomarker fields such as digital biomarker collection. To draw clearer conclusions from future fluid biomarker studies, and to identify the best fluid biomarkers, one approach may be to compare new fluid biomarkers that show promise to the existing gold standard for that fluid biomarker type should one exist (e.g., diagnostic biomarker, prognostic biomarker, etc.). Studies should also evaluate the added benefit of using the new biomarker in conjunction with the current gold standard. While in-depth exploration of a single novel biomarker is important for initial characterization, biomarker studies should move toward incorporating multiple markers. The complexity of ALS and the heterogeneity of the disease among individuals may best be tackled using an array of traits and more sophisticated bioinformatics.

## Conclusion

So far, many fluid biomarker studies have focused on one analyte or occasionally the combined measure of two or more analytes, and few have presented findings that suggest clinical utility at the level of individual patients though neurofilament proteins do show promise in this regard. Going forward, the field will benefit from the combination of clinical data, genetic information, and various biomarker measurements, including fluid, imaging, and digital biomarkers, and the use of bioinformatics/machine learning to establish the best panel of traits and biomarkers for ALS diagnosis, patient care, and clinical trial management.

## Data Availability

All data presented in this review are cited in the referenced articles.

## Abbreviations

ALS: Amyotrophic lateral sclerosis
ALSFRS-R: Revised amyotrophic lateral sclerosis functional rating scale
ASO: Antisense oligonucleotide
AUC: Area under the receiver operating characteristic curve
BEST: Biomarkers, Endpoints, and other Tools
bvFTD: Behavioral variant frontotemporal dementia
C9orf72: C9orf72-SMCR8 complex subunit
c9ALS/FTD: C9orf72-associated amyotrophic lateral sclerosis and frontotemporal dementia
CNS: Central nervous system
CSF: Cerebrospinal fluid
DPR: Dipeptide repeat proteins
ELISA: Enzyme-linked immunosorbent assay
fALS: Familial amyotrophic lateral sclerosis
FDA: U.S. Food and Drug Administration
FTD: Frontotemporal dementia
FUS: FUS RNA binding protein
GBS: Guillain–Barre syndrome
HDGFL2: Hepatoma-derived growth factor like protein 2
Ig: Immunoglobulin
IPN: Inflammatory peripheral neuropathies
MicroRNA: MiRNA
MCI: Mild cognitive Impairment
MND: Motor neuron disease
NAbs: Naturally occurring autoantibodies
ncRNA: Noncoding RNA
NfL: Neurofilament light
NIH: National Institutes of Health
NINDS: National Institute of Neurological Diseases and Stroke
pNfH: Phosphorylated neurofilament heavy
pTDP-43: Phosphorylated TAR DNA-binding protein 43
sALS: Sporadic amyotrophic lateral sclerosis
scFvs: Single chain antibody fragments
SI: Split hand index
SOD1: Superoxide dismutase 1
STMN2: Stathmin 2
SVC: Slow vital capacity
TDP-43: TAR DNA-binding protein 43
TP-181: Phosphorylated tau at threonine 181
TT: Total Tau
UNC13A: Unc-13 homolog A
